# What do doctors want? Incentives to increase rural recruitment and retention in India

**DOI:** 10.1186/1753-6561-6-S1-P5

**Published:** 2012-01-16

**Authors:** Seema Murthy, Krishna Rao, Sudha Ramani, Maulik Chokshi, Neha Khandpur, Indrajit Hazarika

**Affiliations:** 1 Public Health Foundation of India, India

## Introduction

On the occasion the National Rural Health Mission (NRHM) completing its five years, Mr. G B Azad, minister of health and family welfare, declared in his speech “the non-availability of critical human resources continues to be an even larger challenge for which there are no easy solutions”. One of the key priorities of NRHM is to increase availability of human resources in rural India.

Distribution of doctors in India remains highly skewed towards urban areas. Most doctors are employed in the private healthcare sector while many vacancies persist in the government healthcare sector, particularly in rural areas. State governments experience difficulties in staffing rural health centres which in turn undermines various initiatives by NRHM to strengthen rural health services such as making primary health centres and first referral units to work round the clock, and implementation of Indian Public Health Standards.

In this study we examine what doctors expect in order to work in rural areas. We examine career preferences of medical students as well as in-service medical officers working at primary health centres in order to identify incentives that would attract and retain them in rural health services. Our findings inform current practices and policies in regard to recruiting and retaining doctors in rural India.

## Methods

We conducted semi-structured interviews with 68 (23 graduates doing medical internship, 19 postgraduate medical students and 26 in-service doctors) from Andhra Pradesh and Uttarakhand.

Interviews were audio recorded and transcribed for thematic analysis. We clustered stated job attributes by respondents into three broad categories i.e. individual, organisational and contextual attributes. We finalised these attributes through an iterative process and taking a group consensus. We did further rating of each attribute on strength, based on the frequency and force with which the attribute was referred to by respondents.

We also interviewed key policy makers at the state and national level were to get policy perspective on findings from interviews with doctors. We asked policy makers about feasibility of bringing about the changes that doctors were expecting and ranked the attributes accordingly.

## Results

Doctors perceived that the current salaries were not sufficient. They expected increase in salaries; some expected double the current salaries or parity with private healthcare sector.

Many doctors were demotivated by the lack of infrastructure. “What was happening was, we were just looking at the cases and referring them. That was the only thing we were doing” – excerpt from doctors’ interviews. For students, lack of learning opportunities featured prominently. Reservation in post-graduate education for rural medical practitioners was attractive for both, medical interns/students and in-service doctors.

Lack of quality education facilities for children in rural areas was a big deterrent. Security, living facilities, connectivity and proximity to family were among the prominent expectations of doctors to work in rural areas. Many medical students and doctors feared the political interference in rural health service. Medical students viewed rural health services as a stepping-stone for entry into postgraduate education because of the poor image of a rural doctor. Better management, well-defined and transparent transfer policy and increased leaves were seem as important incentives by doctors to serve in rural areas.

Policy makers felt that though salary could not be easily changed, monetary incentives for rural health service could be increased. They felt that Improvement of health centre infrastructure, of in-service learning opportunities, and of living facilities were feasible. They regarded need for better management, transfer and leave policies as not very actionable. They suggested that factors like children's education, connectivity, and security were not actionable as these factors were outside the purview of the health department.

## Discussion

Our study findings suggest that simple solutions like increasing salary are need but not sufficient in recruiting or retaining doctors in rural health services. We need a 'package' of incentives as several factors influence where doctors choose to work. Elements of this package would need to include increase in salary, enhanced opportunities for post-graduate education, better equipped and supplied health facilities, improved living conditions, and clear transfer policies.

It was noteworthy that some critical issues like better management and clear transfer policies were considered to be 'touch me not' issues by policy makers given the political considerations. Yet, all of these are important elements for improving rural recruitment and retention of doctors. NRHM can help addressing the gaps between what doctors expected and what the policy makers felt was feasible. We conclude that bold human resource policies are required to address shortage of doctors in rural India.

